# A Rare Cause of Gastric Outlet Obstruction: A Case Report of Bouveret Syndrome

**DOI:** 10.7759/cureus.112129

**Published:** 2026-07-06

**Authors:** Rachel Pray, Wassem Juakiem

**Affiliations:** 1 Internal Medicine, Keesler Medical Center, Biloxi, USA; 2 Gastroenterology, Keesler Medical Center, Biloxi, USA

**Keywords:** advanced endoscopy, bouveret syndrome, cholecystoduodenal fistula, gallstone ileus, laser lithotripsy

## Abstract

Bouveret syndrome (BS) is a rare phenomenon of gallstone ileus causing gastric outlet obstruction that can present significant challenges in management, given its lack of formal treatment guidelines and endoscopic difficulty. We present a case of an 84-year-old woman with vomiting and abdominal pain who was found to have a large gallstone in the duodenum due to a cholecystoduodenal fistula, as well as findings consistent with acute cholecystitis. She was treated with antibiotics, and gastroenterology and general surgery were consulted. Endoscopy was performed due to high suspicion for BS, revealing a 4-5 cm gallstone impacted in the first portion of the duodenum. Stone removal was extremely challenging, but ultimately 75% of the stone was fragmented using holmium laser lithotripsy, with the remaining fragments passing distally. The patient tolerated the procedure well; antibiotics were continued; and surgery was initially avoided. The patient presented three weeks later with abdominal pain and a small bowel obstruction (SBO) secondary to the remaining stone fragment. General surgery was consulted, and she underwent a laparoscopic small bowel resection with stone retrieval. This case highlights the complex management of BS, which requires advanced endoscopic techniques. However, it is often treated with surgery for definitive management if endoscopy is unsuccessful. Early diagnosis and multidisciplinary collaboration between medicine, gastroenterology, and general surgery are critical to prevent complications in this rare form of gallstone ileus.

## Introduction

Bouveret syndrome (BS) is a form of gallstone ileus caused by a cholecystoduodenal fistula allowing gallstone migration into the duodenum, leading to gastric outlet obstruction [1]. Gallstone ileus comprises only 1-4% of cases of intestinal obstruction, and BS comprises only 1-3% of cases of gallstone ileus [2]. Early diagnosis is critical to prevent complications. Nausea and vomiting are the most common symptoms of BS, followed by abdominal pain or discomfort. Abdominal tenderness is the most commonly reported physical exam finding [3,4]. Diagnosis is made with computed tomography (CT), which shows pneumobilia or cholecystoduodenal fistula in 60% of cases, or esophagogastroduodenoscopy (EGD), which is both diagnostic and therapeutic [2,3]. Management can be complex, often requiring advanced endoscopic techniques or surgery. Early multidisciplinary involvement is essential for optimal outcomes, particularly in refractory or complicated cases [1].

This article was previously presented as a meeting abstract at the 2025 American College of Gastroenterology Meeting on October 27, 2025.

## Case presentation

An 84-year-old woman with a history of hypertension and hyperlipidemia presented to the emergency department with one week of worsening right upper quadrant (RUQ) abdominal pain and persistent vomiting. She also reported decreased appetite and three episodes of non-bloody diarrhea. She denied any fevers, chills, gastrointestinal bleeding, prior abdominal surgeries, or prior biliary endoscopic interventions.

She was hemodynamically stable, afebrile, and had oxygen saturation of 92-96% on 2 liters of supplemental oxygen. The physical exam was significant for an ill-appearing, elderly female with moderate, bilateral upper abdominal tenderness. Bowel sounds were present in all four quadrants, and no pulsatile masses were noted. Lab work revealed a leukocytosis of 13,600 U/L, a hemoglobin of 14.9 g/dL, a potassium of 3.3 mEq/L, and a creatinine of 1.42 mg/dL, which was slightly elevated compared to her baseline creatinine of 0.9 mg/dL.

**Figure 1:**
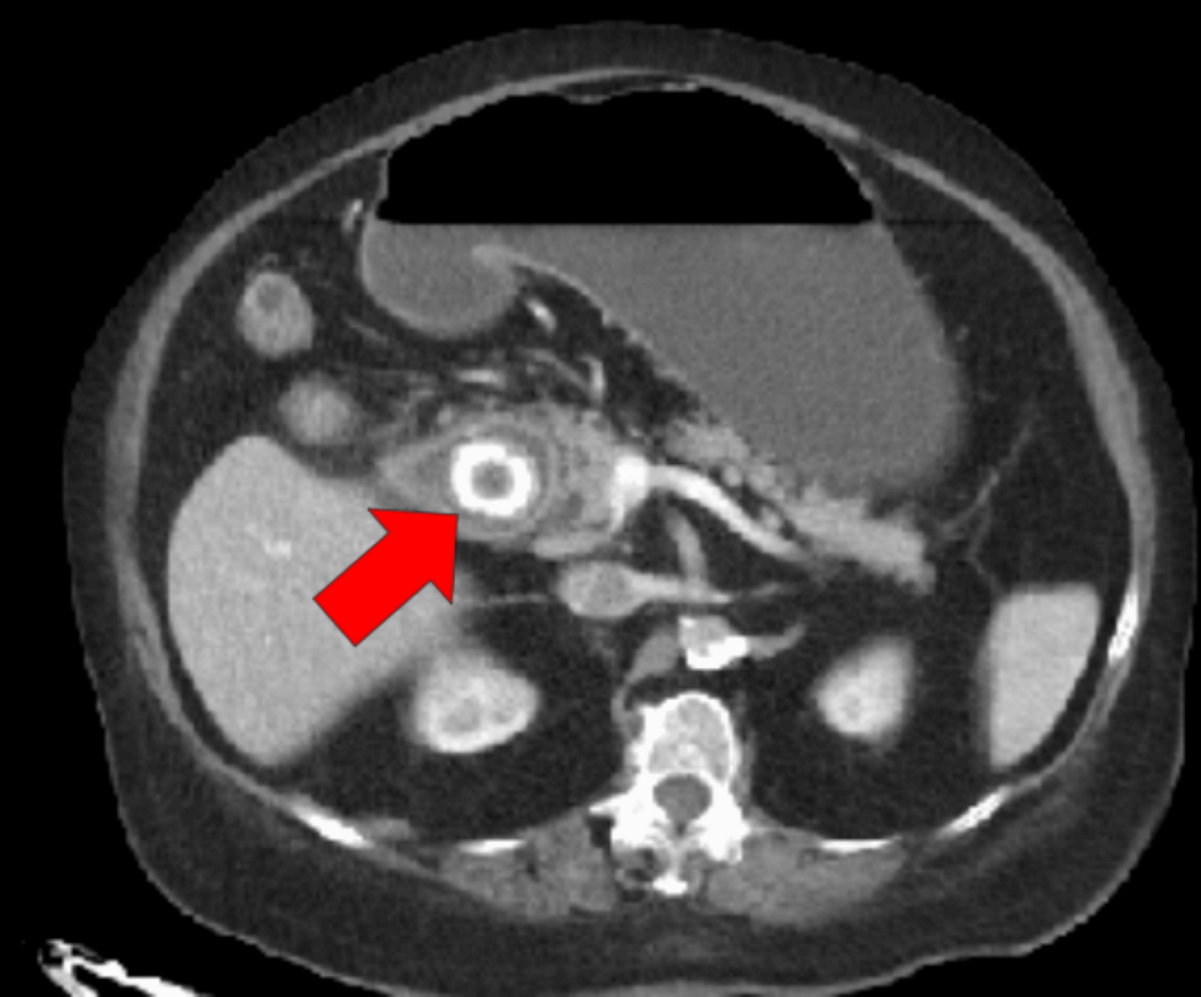
Contrast-enhanced abdominal-pelvic computed tomography (CT) image (axial view). This image demonstrates a large gallstone obstructing the proximal duodenum via a cholecystoduodenal fistula.

**Figure 2:**
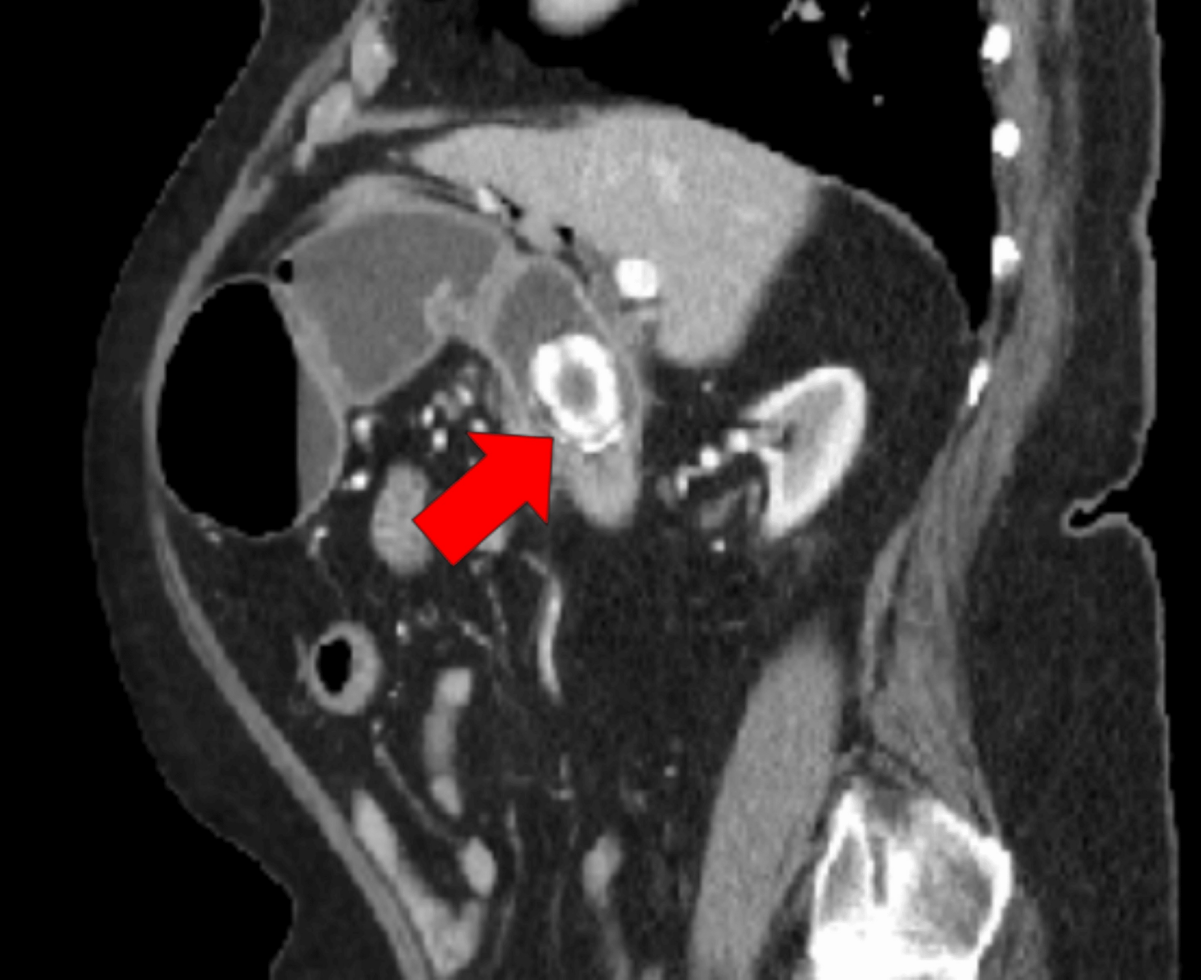
Contrast-enhanced abdominal-pelvic CT image (sagittal view). This image demonstrates dilation in the duodenum secondary to the gallstone. An arrow points to the ectopic gallstone. The cholecystoduodenal fistula is seen between the duodenum and the biliary system. CT: computed tomography.

RUQ ultrasound noted non-specific gallbladder (GB) wall thickening and suboptimal evaluation of the patient’s GB, which appeared contracted. CT abdomen/pelvis with contrast demonstrated circumferential GB wall thickening/enhancement and pericholecystic fat stranding, a cholecystoduodenal fistula, and a 3.2-cm ectopic gallstone in the duodenum (Figures 1-2). Upstream dilation of the stomach and proximal duodenum, consistent with gallstone ileus, was noted. Pneumobilia was also visualized. No biliary ductal dilation was visualized.

In the setting of her RUQ pain, upper abdominal tenderness, leukocytosis, and CT findings demonstrating circumferential GB wall thickening/enhancement and pericholecystic fat stranding, the patient was diagnosed with acute cholecystitis. Piperacillin-tazobactam was started, and general surgery was consulted for potential cholecystectomy. Gastroenterology was consulted for recommendations regarding the ectopic gallstone and cholecystoduodenal fistula.

**Figure 3:**
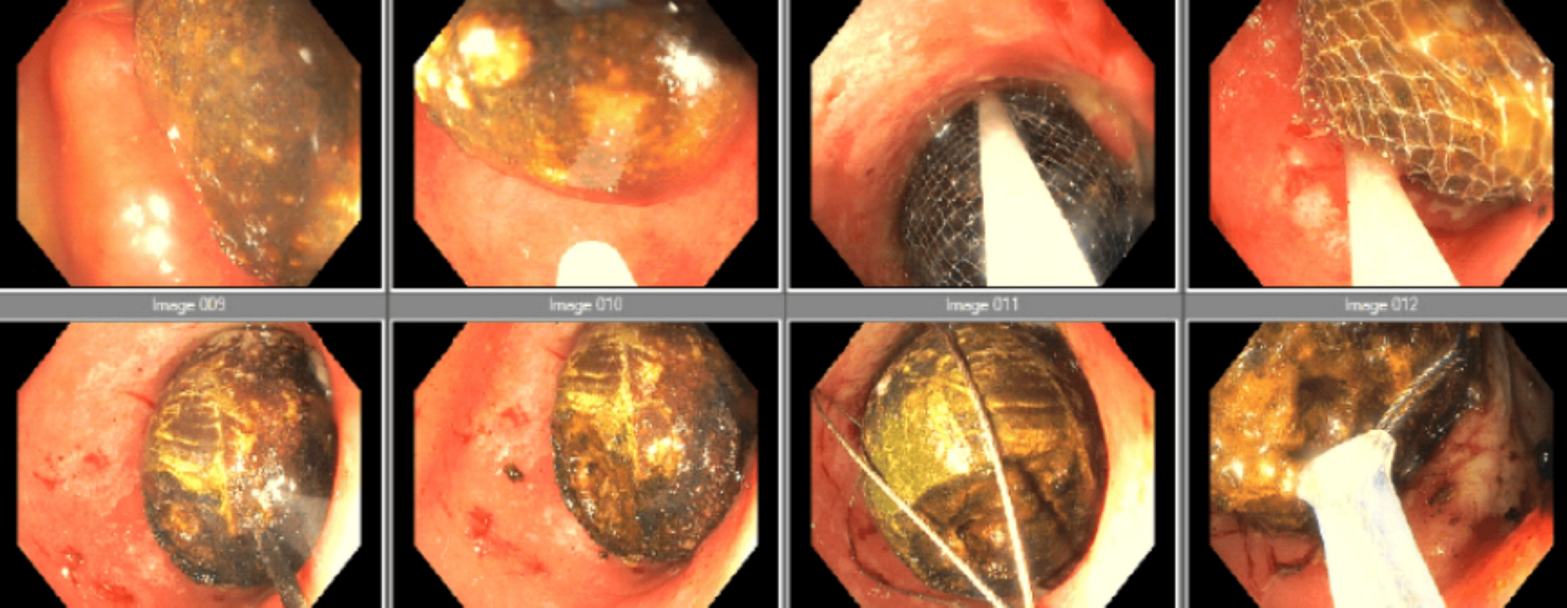
Endoscopic images of gallstone. These images demonstrate the several attempts with various modalities at endoscopic management of the large gallstone impacted in the first portion of the duodenum. An endoscopic stone retrieval basket is seen here.

Gastroenterology evaluated the patient and had high concern for BS, so endoscopic evaluation with removal of the stone and endoscopic retrograde cholangiopancreatography (ERCP) with stent placement was planned. Endoscopy directly visualized the gallstone, revealing its impaction in the first portion of the duodenum. Its size was reported to be between 4 and 5 cm, in comparison to the CT's initial measurement of 3.2 cm. Stone removal proved highly challenging, and multiple techniques were attempted to fragment the stone (Figure 3). Mechanical lithotripsy and electrohydraulic lithotripsy were attempted without success. Ultimately, holmium laser lithotripsy fragmented the stone. Approximately 50% of the stone was removed while the remainder passed distally. The obstruction was resolved without immediate complications, and surgery was initially avoided. Follow-up imaging showed residual fragments within a large duodenal diverticulum. Gastroenterology recommended repeat CT for 2-3 days following endoscopy to evaluate for the remaining 50% of the stone.

The day following endoscopy, the patient denied abdominal pain or vomiting, and abdominal tenderness was no longer present on physical exam. General surgery emphasized the importance of several weeks of healing prior to consideration of fistula takedown or cholecystectomy and recommended continued observation. Repeat imaging was obtained several days after endoscopy, demonstrating stability of the residual gallstone in a large duodenal diverticulum. The patient followed up with gastroenterology outpatient two weeks later and remained asymptomatic. Given the patient's improvement in symptoms and stable location of the gallstone on repeat imaging, gastroenterology and general surgery recommended that further endoscopic intervention or cholecystectomy only occur if the patient developed new symptoms secondary to the residual gallstone.

Three weeks following the initial hospitalization, the patient presented with abdominal pain. Repeat imaging revealed a distal small bowel obstruction (SBO) caused by the residual stone. General surgery was consulted, and the patient was taken to the operating room.

**Figure 4:**
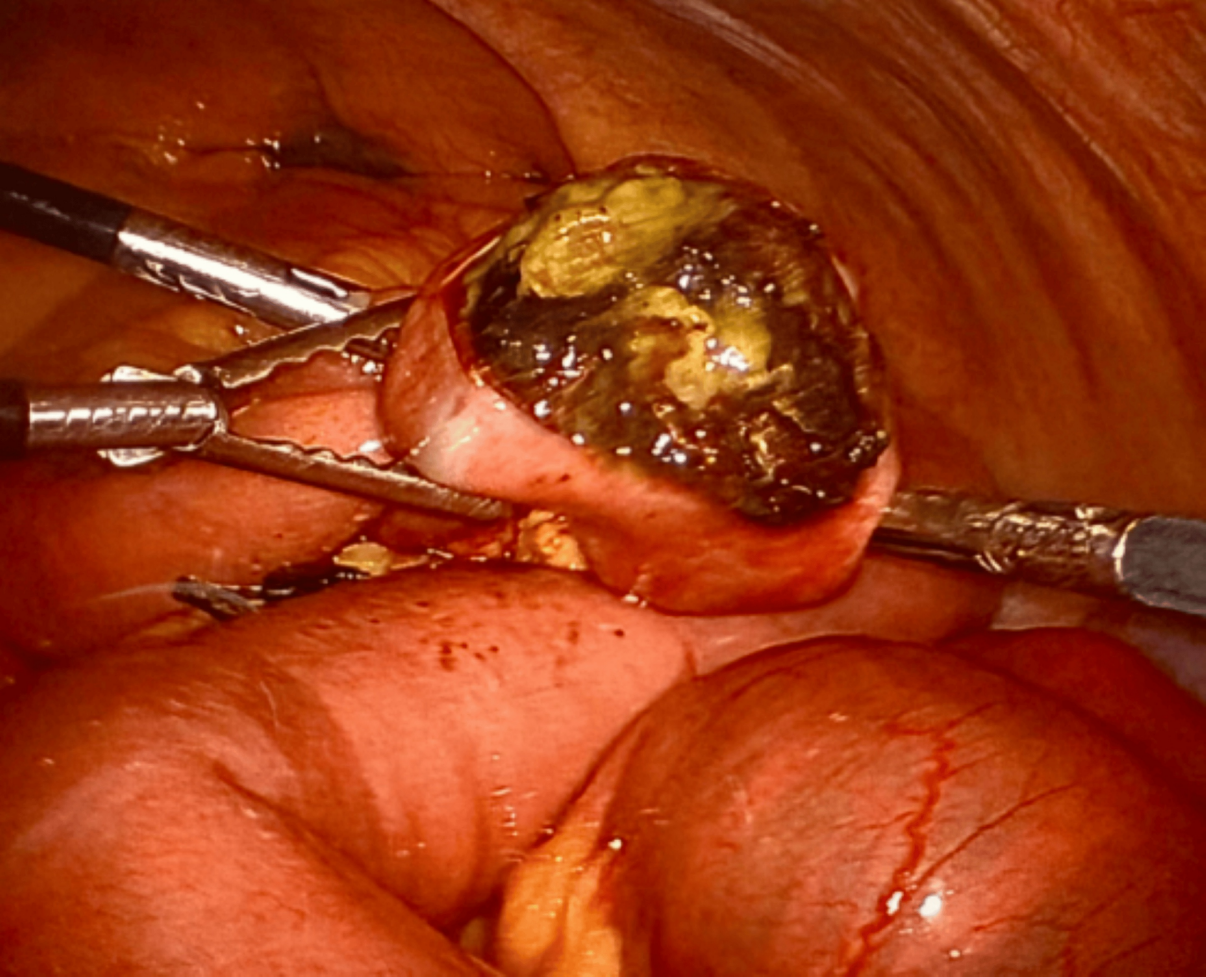
Surgical image of gallstone fragment. Operative findings during diagnostic laparotomy with enterotomy and small bowel resection yielded successful identification and retrieval of the residual shell of a massive gallstone that had migrated distally after initial endoscopy and partial breakup and removal of stone fragments.

The left upper quadrant of the abdomen was insufflated to a pressure of 15 mmHg using a Veress needle technique. An 11 mm left lateral trocar was placed, along with two additional 5 mm trocars in the right abdomen and a 5 mm trocar in the Veress needle site. The dilated proximal bowel was run distally until a palpable mass within the lumen of the bowel was encountered, representing the retained gallstone (Figure 4). The bowel was clamped with an atraumatic bowel clamp, and laparoscopic scissors with electrocautery were used to create an enterotomy on the antimesenteric border of the bowel. The stone was milked from the lumen and placed in a laparoscopic specimen retrieval bag.

An initial attempt was made to laparoscopically close the enterotomy, but the angle of approach proved to be too difficult. This technique was aborted, and focus was placed on retrieving the gallstone. The 11 mm trocar site was enlarged, the fascia was spread with forceps, the laparoscopic specimen retrieval bag was removed from this site, and the gallstone was passed off the field as a specimen. The loop of small bowel was then delivered through the left lateral abdominal incision. A two-layered primary closure was performed at the enterotomy site, the bowel was dunked back into the abdomen, and the left lateral abdominal incision was closed.

Upon intra-abdominal evaluation, there was concern for narrowing of the bowel lumen. This portion of the bowel was then redelivered through the abdominal wall, and a segmental small bowel resection was performed. A stapled side-to-side anastomosis was created using two firings of the 75 mm blue load gastrointestinal anastomosis stapler to divide the bowel and create the common channel. The common enterotomy was closed, the segment of bowel was divided from the mesentery by using a clamping and tying technique, and the segment of bowel was passed off the field as a specimen. The mesenteric defect was closed, and the anastomosis was redelivered into the abdomen. A thorough evaluation for intra-abdominal bleeding was performed. Adequate closure of the mesentery and perfusion of the anastomosis was confirmed.

After successful enterotomy with stone retrieval and small bowel resection with anastomosis, the patient became hypoxic. The procedure was terminated, and takedown of the fistula was not performed. CT pulmonary angiography was obtained, revealing bilateral pulmonary emboli. The patient was started on apixaban, and cardiology and medicine were consulted. She had an uneventful postoperative course.

## Discussion

The rarity of BS limits the amount of available case reports and guidance detailing its management. The CT scan is a well-established diagnostic tool for BS, providing detailed images with information about the location and size of the gallstone, which proves useful for endoscopic planning [1]. EGD is diagnostic of BS and may also be therapeutic; endoscopic retrieval is the first choice in the management of BS and is associated with lower morbidity and mortality compared to surgical management [2,3]. However, it is often extremely technically challenging and may require multiple attempts with various modalities.

Stone fragmentation was achieved in our case with the use of holmium laser lithotripsy after about 240 minutes and attempts with other strategies, including a stone retrieval basket, electrohydraulic lithotripsy, and mechanical lithotripsy. Several other cases also report successful stone fragmentation using laser lithotripsy [3-5]. In a review of treatment outcomes in 110 patients with BS, minimally invasive techniques, including endoscopic retrieval and lithotripsy (laser, mechanical, electrohydraulic, or extracorporeal shock wave), were attempted in 61 cases, yielding an 18% success rate. Laser lithotripsy had the highest percentage of successful cases, obtaining successful retrieval in three out of five cases [3]. In a 67-year-old woman with severe obesity, endoscopic laser lithotripsy for management of a 4 cm by 2 cm duodenal gallstone was planned. Two one-hour sessions of laser lithotripsy achieved some fragmentation of the stone, but it was still unable to be retrieved. Prior to a planned third session, the stone migrated from the duodenum, as seen on repeat CT, and definitive management was accomplished with enterotomy and stone removal [4]. In a 79-year-old woman with a 4 cm gallstone eroding into the duodenum, two sessions of holmium lithotripsy that were 103 minutes and 120 minutes in duration and five days apart successfully fragmented the large stone and treated BS. The patient was monitored in the intensive care unit between sessions due to septic shock, but she tolerated the endoscopic procedure well and was discharged five days later [5]. Laser lithotripsy may offer endoscopists an opportunity for successful stone fragmentation when endoscopically managing BS.

In our case and other cases of BS in which endoscopic interventions were attempted and distal migration of remaining gallstone fragments occurred, repeat CT imaging within 24 hours of endoscopy was useful for identification of stone location and determination of potential surgical management [2,4]. In our patient, repeat CT to evaluate for stone migration was performed directly after endoscopic intervention, and three days following, it demonstrated stable migration to a duodenal diverticulum, and the patient reported significant improvement in her symptoms, so surgery was not pursued. In an 80-year-old woman in whom endoscopic intervention partially fragmented the 4-5 cm stone, CT performed the following day revealed distal migration of the stone to the proximal jejunum, prompting laparoscopic surgical stone retrieval due to concern that the stone would become obstructed in the patient’s known hernia [2]. In another case of partial stone fragmentation following two sessions of holmium laser lithotripsy, CT identified stone migration from the duodenum to the distal ileum after the stone was not found on EGD during the third planned session, prompting surgery for definitive management [4]. Risk factors for gallstone ileus include elderly age and female sex [1-3]. Thus, elderly female patients with BS treated with partial endoscopic stone fragmentation may be more likely to develop complications such as SBO secondary to a residual stone. Larger gallstones may be associated with increased chances of partial fragmentation causing complications, as seen in our case and another case in which the gallstone was reported to be 4 cm or more on endoscopy [2,3]. Complete fragmentation and removal of the gallstone during endoscopy or immediate surgery following partial fragmentation of a stone on endoscopy would likely prevent future ileus caused by residual stone fragments. However, the decision to pursue surgery is individualized and requires thoughtful consideration, including assessing a patient’s stability, comorbidities, risk of potential complications, and patient preferences. No well-established management guidelines exist for monitoring of residual stone fragments or prevention of SBO in patients with BS; however, repeat CT imaging may be a valuable tool for monitoring and assessing surgical necessity.

While endoscopic retrieval is the first choice in management of BS, it may not be technically feasible, making surgery necessary for definitive management. Commonly, surgical management includes enterolithotomy or gastrotomy, with or without cholecystectomy and fistula repair. In patients with BS that are elderly and have multiple comorbidities, a more restrictive surgical strategy with stone extraction and no fistula repair has been recommended to avoid additional operative complications. Two elderly patients with BS and multiple comorbidities that previously failed endoscopic intervention and underwent surgery for stone extraction without fistula repair tolerated their procedures well without significant postoperative complications [6]. In our case, general surgery and gastroenterology were simultaneously consulted on admission, and the surgeon that performed the patient’s small bowel resection on the second admission was familiar with our patient’s case. A diagnostic laparoscopy with enterotomy, stone retrieval, and small bowel resection was performed in our patient without postprocedural complications. In the aforementioned review of 110 cases with treatment outcomes, the most common surgical complications were bile leak, duodenal leak, and death [3]. Given these increased risks with surgery, early multidisciplinary collaboration with surgical colleagues is important to decrease delays in care and to prevent further complications such as infection, dehydration, or perforation [1]. Clinicians and patients should be aware that while endoscopy is the safer option, surgery will likely be necessary for the treatment of BS.

## Conclusions

This case highlights the challenging management required for a rare form of gallstone ileus in an elderly female. Given the significant technical difficulty of BS, even a skilled endoscopist may not be able to successfully fragment these gallstones. Several endoscopic techniques were attempted in our case, but holmium laser lithotripsy achieved partial stone fragmentation, initially avoiding surgery. When our patient presented three weeks later with an SBO secondary to the retained gallstone fragment, definitive management of BS was achieved with surgery. CT imaging proved a valuable tool to determine the location of the gallstone before and after procedures. The three pillars of our patient’s care that ultimately resulted in a safe, successful outcome were proper medical management with antibiotics and early consultations with subspecialists, advanced endoscopic intervention, and surgical management.
